# *In Vitro* Protective Effects of* Lycium barbarum* Berries Cultivated in Umbria (Italy) on Human Hepatocellular Carcinoma Cells

**DOI:** 10.1155/2016/7529521

**Published:** 2016-11-14

**Authors:** M. R. Ceccarini, S. Vannini, S. Cataldi, M. Moretti, M. Villarini, B. Fioretti, E. Albi, T. Beccari, M. Codini

**Affiliations:** ^1^Department of Pharmaceutical Science, University of Perugia, Via Fabretti 48, 06122 Perugia, Italy; ^2^Department of Chemistry, Biology and Biotechnology, University of Perugia, Via Elce di Sotto 8, 06123 Perugia, Italy

## Abstract

*Lycium barbarum* is a famous plant in the traditional Chinese medicine. The plant is known to have health-promoting bioactive components. The properties of* Lycium barbarum* berries cultivated in Umbria (Italy) and their effect on human hepatocellular carcinoma cells (HepG2) have been investigated in this work. The obtained results demonstrated that the* Lycium barbarum* berries from Umbria region display high antioxidant properties evaluated by total phenolic content and ORAC method, on hydrophilic and lipophilic fractions. Moreover, on HepG2 cell line* Lycium barbarum* berries extract did not change cell viability analyzed by MTT and Trypan blue exclusion assay and did not induce genotoxic effect analyzed by comet assay. Furthermore, it was demonstrated, for the first time, that the berries extract showed a protective effect on DNA damage, expressed as antigenotoxic activity* in vitro*. Finally,* Lycium barbarum* berries extract was able to modulate the expression of genes involved in oxidative stress, proliferation, apoptosis, and cancer. In particular, downexpression of genes involved in tumor migration and invasion (CCL5), in increased risk of metastasis and antiapoptotic signal (DUSP1), and in carcinogenesis (GPx-3 and PTGS1), together with overexpression of tumor suppressor gene (MT3), suggested that Umbrian* Lycium barbarum* berries could play a protective role against hepatocellular carcinoma.

## 1. Introduction

The use of medicinal and edible plants was widely distributed from ancient times to today in Asian countries. During the last twenty years much attention has been paid to plants as novel alternative therapeutic agents and/or as support to the traditional medicine in Europe and North America [1]. The Solanaceae, one of the largest and most important families of flowering plants, includes* Lycium barbarum* species that are recorded in the Chinese Pharmacopoeia [2]. The plant is commonly called Goji [1] and active molecules have been isolated from seeds, fruits, and leaves of* Lycium barbarum*.* In vitro* and* in vivo* studies displayed antihypertensive, antihyperglycemic, antitumor, antihyperlipidemia, and anti-Alzheimer activities of* Lycium barbarum* berry (LBB) extract [3].

LBB extract contains high level of health-promoting bioactive components including polysaccharides, flavonoids, and carotenoids [1–4]. Polysaccharides have been considered the major ingredients responsible for the biological activities of LBB extract. Traditional Chinese medicine considered LBBs to have the ability to maintain the function of eyes and strengthen the activity of liver, kidneys, and lungs [3]. In addition, LBB extract has been historically used as anti-inflammation and antiaging agent for thousands of years [5]. In fact, Oh et al. [6] demonstrated that LBB extract has inhibitor effect on proinflammatory mediator production in lipopolysaccharide-stimulated RAW 264.7 cells via blockade on the MAPKs and NF-*κ*B pathways. Additionally, in the last few years, LBBs have been described to modulate the aging by acting on cp53-mediated pathway [7] and on the resistance to the generation of lipid peroxide and other substances, which damage cell membrane lipid [8]. LBB extract has been also described for its immune enhancing [9], antioxidant and anticancer [3], and hepatoprotective and neuroprotective [10] properties. Moreover, it has been highlighted that LBB extract has hypoglycemic and hypolipidemic effects by reducing significantly blood glucose levels and serum total cholesterol and triglyceride concentrations [11]. Interestingly it has been reported a protective effect of LBBs against doxorubicin-induced cardiotoxicity through antioxidant-mediated mechanisms. In particular LBBs significantly prevents the loss of myofibrils and improves the heart function of the doxorubicin-treated rats [12]. Finally Wang et al. [13] demonstrated that sulfated LBB polysaccharides significantly inhibit the infectivity of Newcastle disease virus to chicken embryo fibroblast.

The original habitat of* Lycium barbarum* is probably located in the warm regions in Mediterranean area and Southwest and Central Asia [1]. Recently the plant adaptation to different environments has been reported in a study of* Lycium barbarum* cultivation in Tuscany (Italy) [14].

In this paper we report for the first time that LBBs, cultivated in Umbria (Italy), have very good antioxidant properties, evaluated by two different methods. Moreover, LBBs are able to protect* in vitro* HepG2 cells from genotoxicity induced by 1,2,4-benzenetriol (BT) and stimulate MT3 tumor suppressor gene, suggesting that LBBs could play a specific role in maintaining cell health.

## 2. Materials and Methods

### 2.1. Materials

LBBs cultivated in Umbria were provided by Impresa Agricola of Gianluca Bazzica, Foligno (Italy); commercial LBBs were bought in pharmacy, Perugia (Italy). Human Caucasian hepatocyte carcinoma HepG2 cells were purchased from Istituto Zooprofilattico Sperimentale della Lombardia e dell'Emilia Romagna “Bruno Ubertini” (Brescia, Italy). Eagle's Minimum Essential Medium (MEM), L-glutamine, trypsin, and ethylenediaminetetraacetic acid disodium and tetrasodium salt (EDTA) were from Microtech Srl (Pozzuoli, NA, Italy). Fetal Bovine Serum (FBS) and penicillin-streptomycin were from Thermo Fisher Scientific (Waltham, MA, USA). Antibiotics, sodium pyruvate, and Dulbecco's phosphate-buffered saline pH 7.4 (PBS) were purchased from Invitrogen Srl (Milan, Italy). Dimethyl sulfoxide (DMSO), ethanol, hydrochloric acid, sodium chloride, and sodium hydroxide were purchased from Carlo Erba Reagenti Srl (Milan, Italy). 6-Hydroxy-2,5,7,8-tetramethylchroman-2-carboxylic acid (Trolox), Trypan blue solution 0.4%, acridine orange, 4′,6-diamidine-2′-phenylindole dihydrochloride (DAPI), ethidium bromide, low- and normal-melting-point agarose (LMPA and NMPA), 1,2,4-benzenetriol (BT), staurosporine, tris(hydroxymethyl)aminomethane (Tris), Triton X100, valinomycin, Folin-Ciocalteu, 2,20-azobis (2-methylpropionamide) dihydrochloride (AAPH), and 3-[4,5-dimethyl-2-thiazolyl]-2,5-diphenyl-2-tetrazolium bromide (MTT) were obtained from Sigma-Aldrich Srl (St. Louis, MO, USA).

### 2.2. Preparation of LBB Extract

Berries from* Lycium barbarum* were collected from 3-year-old trees growing in Foligno, Umbria Region, Italy (42°55′26.9′′ north, 12°39′29.2′′ east, and altitude 230 m). In this area, the climate is warm and temperate according to Cfa class in Köppen and Geiger classification [15]. The average annual temperature and mean annual rainfall are 14.3°C and 706 mm, respectively. The global solar radiation (on the ground) is 5235 MJ/m^2^ (dates from ENEA, http://clisun.casaccia.enea.it/). Commercial LBBs were used as controls. Umbrian and commercial LBBs (1 g) were homogenized in physiological solution (10 mL) with Ultra Turrax T25 Basic homogenizer (Ika Labortechnick, Staufen, Germany) at room temperature for 1 min followed by centrifugation at 3150 ×g for 30 min and the supernatant was used for all experiments.

### 2.3. Total Phenolic Content (TPC)

The total phenolic content (TPC) of commercial and Umbrian LBB extract was determined using the Folin-Ciocalteu colorimetric method described by Rashidinejad et al. [16] with modifications [17]. Gallic acid stock solution (5 mg/mL) and working standard concentrations of 0, 10, 25, 50, 100, 250, and 500 *μ*g/mL were prepared in deionized water. The Folin-Ciocalteu procedure consisted of transferring 20 *μ*L standard or sample into 4-5 mL borosilicate tube, followed by addition of water (1.58 mL) and Folin-Ciocalteu reagent (100 *μ*L). After mixing the samples, 300 *μ*L of 20% Na_2_CO_3_ was added and the samples mixtures were kept for 30 min at 40°C. The total phenols were determined at 765 nm. Total phenol values are expressed in terms of gallic acid equivalent (GAE), which is a common reference compound.

### 2.4. Antioxidant Assay by Oxygen Radical Absorbance Capacity (ORAC)

The antioxidant capacity of Umbrian LBB extract was determined using the ORAC method [18]. The hydrophilic and lipophilic fractions were extracted according to Prior et al. [19]. A duplicate extraction was performed for each sample and used to evaluate the lipophilic (L-ORACFL) and hydrophilic ORACFL (H-ORACFL) values [19]. Evaluations of the lipophilic and hydrophilic ORACFL in the LBBs samples were performed separately, and the total antioxidant capacity (TAC) was calculated by adding the L-ORACFL and H-ORACFL values [20]. The ORACFL assays were carried out on a FLUOstar OPTIMA microplate fluorescence reader (BMG LABTECH, Offenburg, Germany) at an excitation wavelength of 485 nm and an emission wavelength of 520 nm. The procedure was based on the method of Zulueta et al. [21] with slight modifications. Briefly, 2,20-azobis (2-methylpropionamide) dihydrochloride (AAPH) was used as a peroxyl radical generator, Trolox was used as a reference antioxidant standard, and fluorescein was used as a fluorescent probe. The data are expressed as micromoles of Trolox equivalents (TE) per gram of sample (*μ*mol TE/g).

### 2.5. Cell Culture and Treatments

HepG2 cells were grown in monolayer cultures in 25 cm^2^ tissue flasks, with MEM supplemented with 10% heat-inactivated FBS, 1 mmol/L of sodium pyruvate, 2 mM of L-glutamine, and antibiotics (100 U/mL penicillin, 100 *μ*g/mL streptomycin). The cells were maintained in a cell incubator at 37°C in a humidified atmosphere containing 5% CO_2_. When the cells reached 80–90% of confluence, the routine culture medium was aspirated and the HepG2 cells were washed with PBS 1X. The cells were then harvested by 0.05% trypsin in 0.02% Na_4_EDTA for 5 min at 37°C and suspended in 1 : 3 supplemented growth medium to be maintained in the exponential growth phase.

### 2.6. Cell Viability

Cell viability was tested by MTT and Trypan blue exclusion assay.

#### 2.6.1. MTT Assay

Cellular viability was assessed by the reduction of MTT to formazan [22]. HepG2 cells were seeded onto 96-well plate at a density of 1 × 10^4^ cells/well with MEM complete medium. After 24 h in each well culture medium was replaced with fresh complete medium containing different concentrations (400, 600, 800, 1000, 1200, 1400, 1600, 1800, 2000, 2200, 2400, 2600, and 2800 *μ*g/mL) of Umbrian LBB extract and incubated for additional 24 h. Then, MTT reagent was dissolved in PBS 1x and added to the culture at 0.5 mg/mL final concentration. After 3 h incubation at 37°C, the supernatant was carefully removed and formazan salt crystals were dissolved in 200 *μ*L DMSO added to each well. The absorbance (OD) values were measured spectrophotometrically at 540 nm using an automatic microplate reader (Eliza MAT 2000, DRG Instruments, GmbH). Each experiment was performed two times in quadruplicate. Cell viability was expressed as a percentage relative to that of the control cells set at 100%.

#### 2.6.2. Trypan Blue Exclusion Assay

Trypan blue was performed according to Srivastava et al. [23] with modifications. Cytotoxicity using the Trypan blue exclusion assay was measured using a Countess™ (Invitrogen Srl, Milan, Italy) automated cell counter. Briefly, 50 *μ*L of HepG2 cell suspensions was mixed with equal volumes of 0.4% Trypan blue and loaded onto a Countess cell counting chamber slide. The instrument is equipped with a camera that acquires images from cell samples on the chamber slide, and the image analysis software automatically analyzes acquired cell images and measures cell count and viability.

### 2.7. Comet Assay

Cells, for genotoxic and antigenotoxic assays, were analyzed by comet assay [24]. For genotoxicity testing, HepG2 cells were seeded onto 6-well plate at a density of 1 × 10^5^ cells/well with MEM complete medium. After 48 h, in each well culture medium was replaced with fresh complete MEM containing different concentrations (200, 600, 1000, 1400, and 1800 *μ*g/mL) of Umbrian LBB extract and incubated for 4 h. Negative (MEM) and positive 100 mM of 1,2,4-benzenetriol (BT) controls were included in each experimental set [25]. Each experimental set was repeated at least 3 times. For antigenotoxicity testing, HepG2 cells were cultured for 15 days in the presence of 1800 *μ*g/mL of LBB extract, added to the medium as a nutritional supplement. After the treatment, LBBs extract was removed from the medium to avoid scavenger effects. The HepG2 cells were divided into two groups, the first one (negative control) grown only in complete medium (MEM) and the second one grown with MEM added with LBB extract. Both groups were used to perform comet assay and incubated 4 h with only MEM or with 100 mM of BT. Each experimental set was repeated at least 3 times [26].

For both experiments cells were collected by centrifugation at 70 g for 8 min at 4°C and then processed in the comet assay under alkaline conditions (lysis at pH 10, unwinding and electrophoresis at pH > 13). The comet assay was carried out basically following the original procedure [24], with minor modifications [27] using the double-spot system. Briefly, cell pellets were gently resuspended in 0.7% LMPA in PBS maintained at 37°C. Then, the cell suspensions were rapidly layered onto agarized microscope slides. After the gels were allowed to solidify, the slides were immersed in cold, freshly prepared cellular lysing solution (2.5 M NaCl, 100 mM Na_2_EDTA, 10 mM Tris-HCl; pH 10; and 1% Triton X100 added just before use) overnight at 4°C. After the membranes lysis, the slides were placed in a horizontal electrophoresis box (HU20, Scie-Plas, Cambridge, UK) filled with a freshly prepared electrophoresis solution (10 mM Na_4_EDTA, 300 mM NaOH; pH > 13). After 20 min of preelectrophoresis to allow DNA unwinding and expression of alkali-labile damage, electrophoresis runs were performed in an ice bath for 20 min by applying an electric field of 1 V/cm and adjusting the current to 300 mA (Power Supply PS250, Hybaid, Chesterfield, MO, USA). The microgels were then neutralized with 0.4 M Tris-HCl buffer (pH 7.5). For scoring, the slides were stained with 50 *μ*L of EtBr (20 *μ*g/mL). The comets in each microgel were analyzed (blind), at 200x magnification, with an epifluorescent microscope (BX41, Olympus Co., Tokyo, Japan) under a 100 W high-pressure mercury lamp (HSH-1030-L, Ushio Inc., Tokyo, Japan), using appropriate optical filters (excitation filter 510–550 nm and emission filter 590 nm). The microscope, equipped with a high sensitivity black and white CCD camera (PE2020, Pulnix Europe Ltd., Basingstoke, UK), was connected to a computerized analysis system (“comet assay III,” Perceptive Instruments, Suffolk, UK). The tail intensity, that is, percent of fluorescence migrated in the comet tail, which is considered to be the most useful parameter system [28], was used to evaluate DNA damage. A total of 100 randomly selected comets (50 cells/replicate spot) were evaluated for each experimental point. For each independent test, the median tail intensity of 50 cells/spot was assessed and the average of 2 replicated spots was calculated as a summary statistic [29].

### 2.8. Acridine Orange and DAPI Staining

In order to determine cell viability, the same samples, which contained cells in suspension, used for comet assay were mixed with a solution of acridine orange (30 *μ*g/mL) and DAPI (100 *μ*g/mL). Acridine orange is necessary to stain the entire population of cells, while DAPI is used to stain nonviable cells. Briefly, for each sample 5 *μ*L of mixture of dyes was added to 95 *μ*L of cell suspension. Then the samples were immediately loaded into the NC-Slides A8 and read with the NucleoCounter NC-3000 analysis system (ChemoMetec A/S, Denmark). The system recognizes and counts all cells (green fluorescence) and the nonviable cells (blue fluorescence), subtracting the latter value to the first automatically and then returning the data related to the viability of each sample.

### 2.9. PCR-Array Analysis

HepG2 cells cultured in the absence or presence of Umbrian LBB extract were used for total RNA extraction performed by using RNAqueous®-4PCR kit (Ambion Inc., Austin, Texas) as previously reported [30]. Samples were treated with RNAse-free DNase to prevent amplification of genomic DNA. Samples were dissolved in RNAse-free water and total RNA was quantified by measuring the absorbance at 260 nm (*A*_260_). The purity of RNA was evaluated by using the *A*_260_/*A*_280_ ratio. *A*_260_/*A*_230_ ratio also was used as indicator of chemical contaminants in nucleic acids. The extracted RNA was immediately frozen and maintained at −80°C. Before cDNA synthesis, the integrity of RNA was confirmed by denaturing electrophoresis in TAE 1.2% agarose gel [31]. cDNA was synthesized using 1 *μ*g total RNA for all samples by High-Capacity cDNA Reverse Transcription kit (Applied Biosystems, Foster City, CA, USA) under the following conditions: 50°C for 2 min, 95°C for 10 min, 95°C for 15 sec, and 60°C for 1 min for 40 cycles. RTqPCR was performed using Master Mix TaqMan® Gene Expression and 7.300 RT-PCR instrument (Applied Biosystems), targeting genes in TaqMan Array 96-Well Plate P/N: 4414250.

### 2.10. Statistical Analysis

Data were reported as the mean ± SD of experiments conducted in triplicate. The significance of treatment was analyzed using the Student *t*-test (*p* value was <0.001).

## 3. Results and Discussion

**Figure 1:**
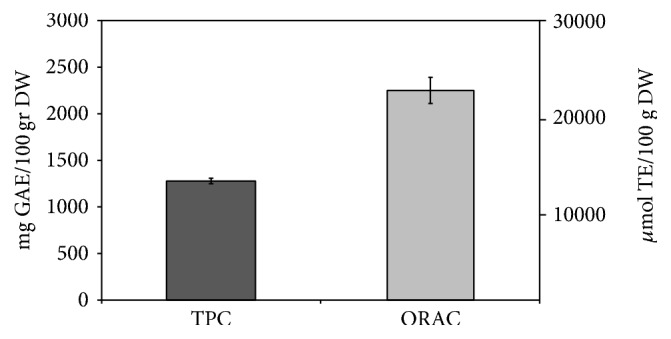
Antioxidant properties of LBBs. Data for total phenolic content (TPC) are referred to the left ordinate, while data for antioxidant capacity (ORAC) are referred to the right ordinate. Results are expressed as mean ± SD of three independent experiments.

*Lycium barbarum* cultivated in Umbria grows up to 2 meters similarly to Chinese one [1]. The plant produces a bright orange-red, oval berry 1.5 cm long and possesses a sweet taste. In the traditional East Asian medicine the LBB extract is known to have beneficial effects for the health, thanks to their antioxidant properties [32]. Thus, we first evaluated the total phenolic content (TPC) of LBBs cultivated in Umbria in order to make a comparison with commercial LBBs produced in Asia. TPC value is 1278.247 ± 29.60 mgGAE/100 g dry weight (DW), using 80% ethanol for the extraction (Figure 1). It has been reported that dehydrated LBBs had the TPC value of 351 ± 7.25 mgGAE/100 g, performing the extraction with 80% methanol [33]. The influence of the solvent, used for the extraction on the TPC value, has been previously investigated and it was found a reduction of 1.3-fold or 2.2-fold using methanol instead of ethanol [34, 35]. In any case, also taking into account the variability due to different method of extraction, the Umbrian LBBs have a TPC higher than commercial one. To confirm these result 80% ethanol extract of commercial LBBs was prepared. The results show a TPC value of 712.01 ± 29.12 mgGAE/100 g, similar to that reported in the literature [23], confirming the highest TPC of Umbrian LBBs. It is difficult to establish exactly the reason of this difference, but we can hypothesize that the climate, the season, and therefore the hours of sunshine could contribute positively to the result of TPC.

The Umbrian LBB extract exhibits antioxidant activity value of 22507.03 ± 1402.02 *μ*mol TE/100 g DW with ORAC method (Figure 1), whereas the value of commercial LBB extract is 26502 ± 3807 *μ*mol TE/100 g DW. Thus, the values obtained for LBBs cultivated in Umbria and commercial LBBs, in terms of antioxidant activity, were similar despite the different TPC. It is possible to conclude that both TPC and antioxidant activity are very high in LBBs cultivated in Umbria ground. These results do not mean that TPC and ORAC are directly correlated because TPC evaluates only the polyphenol antioxidant properties, whereas ORAC indicates the total antioxidant properties.

**Figure 2:**
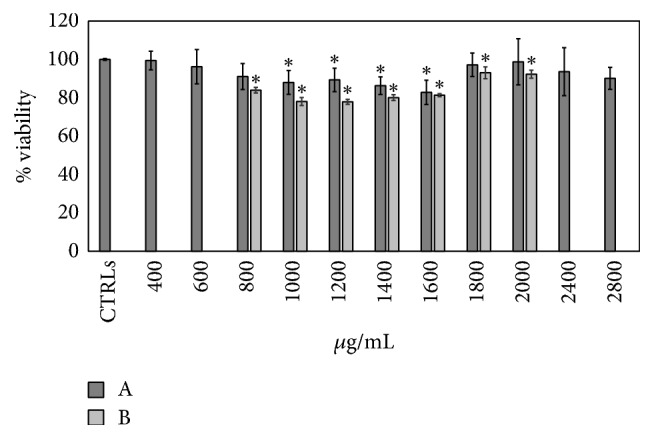
Effect of LBB extract on cell viability. HepG2 cells are treated for 24 h with different concentrations of LBBs (from 400 *μ*g/mL to 2800 *μ*g/mL). Cell viability is measured by MTT assay (A) and by Trypan blue exclusion assay (B). The values are reported as % viability of the control sample set at 100%. Data are expressed as mean ± SD of four independent experiments (^*∗*^ *p* < 0.001).

Recently, LBBs have been described to have apoptotic and antiproliferative effects on cancer cells* in vitro* and* in vivo* [3]. Based on these results, we investigated the cytotoxicity, genotoxicity, and antigenotoxicity of Umbrian LBB extract in HepG2, human hepatocellular carcinoma cells. This cell line has been chosen for its high degree of morphological and functional differentiation* in vitro* and also because it is a suitable model to study drug and plant metabolites targeting* in vitro* [36–38]. MTT assay has been used to test cell viability at different concentration of LBB extract (400, 600, 800, 1000, 1200, 1400, 1600, 1800, 2000, 2200, 2400, 2600, and 2800 *μ*g/mL) after 24 h of culture (Figure 2). With low concentrations (400–800 *μ*g/mL) and with high concentrations (1800–2800 *μ*g/mL) of LBB extract, the cell viability did not change in comparison with control cells (CTRLs). At 1000, 1200, 1400, and 1600 *μ*g/mL concentrations the cell viability was reduced by 12%, 11%, 14%, and 18%, respectively, indicating that highest inhibitory effect of LBBs was at 1600 *μ*g/mL concentration. As shown in Figure 4 the highest concentrations (from 1800 to 2800 *μ*g/mL) have a very important standard deviation. Given the nature of cell type, that is, an immortalized cell line, the variability is normal and the standard deviation conforms to the experimental system. Anyway the cell viability is more than 80% in overall concentration used. The behavior of the LBBs assays by Trypan blue exclusion test appears similar to that obtained by MTT assay (Figure 2). The vitality percentage with Trypan blue assay evaluated at critical concentration (from 800 to 2000 *μ*g/mL) is lower than the vitality percentage obtained with MTT assay according to previous observations [39].

The increase of cell viability at concentration up to 1600 *μ*g/mL could be explained by the nonlinear dose-responses of plants and other natural products [40]. Gan et al. [41] demonstrated that 10 mg/kg dose of Chinese LBBs was more effective than 5 and 20 mg/kg doses in the reduction of sarcoma weight and in improving the immune system in the mice.

**Figure 3:**
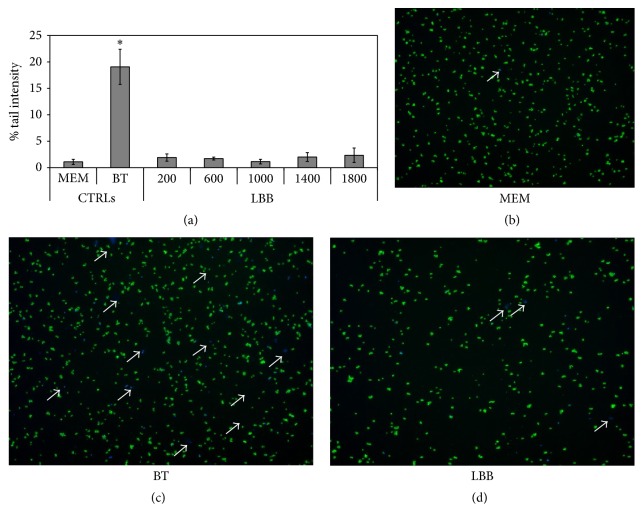
Effect of LBB extract in HepG2 cells. (a) Genotoxic effect, determined by comet assay, after 4 h of treatment with different concentrations of LBBs (200, 600, 1000, 1400, and 1800 *μ*g/mL). MEM is used as negative control, and 1,2,4-benzenetriol (BT), known to induce oxidative damage to DNA, is used as positive control. Results are expressed as mean ± SD of three independent experiments (^*∗*^ *p* < 0.05); (b) acridine orange and DAPI staining for negative control (MEM); (c) same staining for positive control (100 mM of 1,2,4-benzenetriol, BT); (d) same staining for LBB extract (1800 *μ*g/mL). The arrows indicate the death cells stained in blue with DAPI.

**Figure 4:**
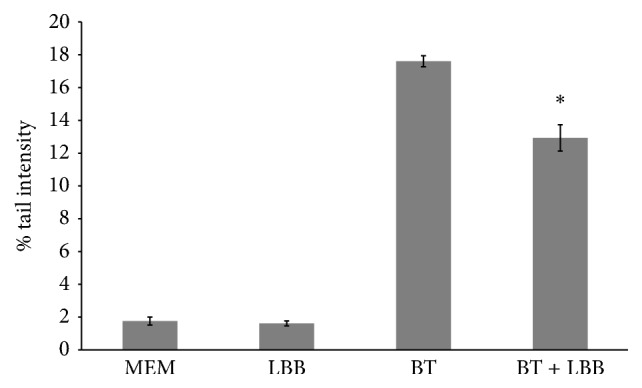
Antigenotoxic effect of LBB extract (1800 *μ*g/mL for 15 days) and the cotreatment for 4 h with BT (1,2,4-benzenetriol) that induced DNA damage in HepG2 cells. MEM is used as negative control, and 1,2,4-benzenetriol (BT), known to induce oxidative damage to DNA, is used as positive control. Each result is expressed as the mean ± SD of three independent experiments (^*∗*^ *p* < 0.001).

Thus, we demonstrated that Umbrian LBBs weakly influence HepG2 cell viability in a dose depending manner but without any cytotoxic effect at all concentration considered. The subsequent objective was to test the potential genotoxic effect on HepG2 cell line at different concentrations of LBB extract (200, 600, 1000, 1400, and 1800 *μ*g/mL) after 4 h of treatment. The reason for selecting this exposure time (4 h) is to avoid the initiation of DNA repair events that would result in an underestimation of the damage. Untreated cells, used as negative control, show a tail intensity of 1.1 ± 0.24% whereas the positive control, 100 mM of 1,2,4 benzenetriol (BT), reveals a significant high percentage of tail intensity of 19.07 ± 1.66% (Figure 3(a)). BT is a metabolite of benzene which leads to the formation of numerous free radicals inside the cells, able to cause oxidative damage to DNA [25]. The results obtained with all LBB concentrations are similar to negative control sample indicating the absence of genotoxicity (Figure 3(a)). This result is very important because it is the first time that genotoxicity has been tested on LBB extract and it should be evaluated in all type of phytoextracts. Finally the LBB highest concentration (1800 *μ*g/mL) was used to confirm the viability of cells using acridine orange and DAPI staining after 4 h of treatment just before proceeding to the comet assay. Only the sample treated with BT showed a high number of positive cells to DAPI, colored in blue (Figure 3(c)). This result further confirms that Umbrian LBBs have no genotoxic effect on HepG2 cell line (Figure 3(d)). The absence of cytotoxicity and genotoxicity in LBBs extract is of fundamental importance for the use of this plant extract in the diet as a strong antioxidant. The positive actions of LBB extract in disease prevention are now mainstream and commercial health claims being made are subject to regulation in most countries. To this end, for the first time, the antigenotoxic effect of LBB extract has been tested* in vitro* using the HepG2 cell line. The HepG2 cells were cultured for 15 days in the presence of 1800 *μ*g/mL of LBB extract, previously used for genotoxic assay. The recommended dosage of LBBs in human varies between 5 g and 12 g. If you considered a subject of 70 kg medium weight with about 5 L of blood, the inhibitor concentrations we used range from 5 g to 8 g. Since 8.5 g is medium recommended dosage [1], we used 1800 *μ*g/mL concentration corresponding to about 9 g to test if, despite no changes in viability and toxicity, it could induce changes in genes expression on HepG2 cells. In Figure 4 the DNA damage induced by BT is reported when HepG2 cells were pretreated for 15 days with LBBs. Untreated cells, used as negative control, show a tail intensity of 1.43 ± 0.34% and the cells treated with LBB extract for 15 day have a tail intensity of 1.28 ± 0.15%. This result confirms the absence of genotoxicity induced by LBB extract not only for 24 h (Figure 3(a)), but also after 15 days of treatment. The positive control, 100 mM BT, reveals a tail intensity of 17.59 ± 0.33%, but the cells preexposed to LBBs show a tail intensity of 12.45 ± 0.84%. It means that the LBB extract is able to reduce significantly the DNA damage of 29.3%, if we consider the positive control as a 100% of DNA damage. This finding is of great importance for the use the LBB extract in the diet or for the production of functional food.

**Figure 5:**
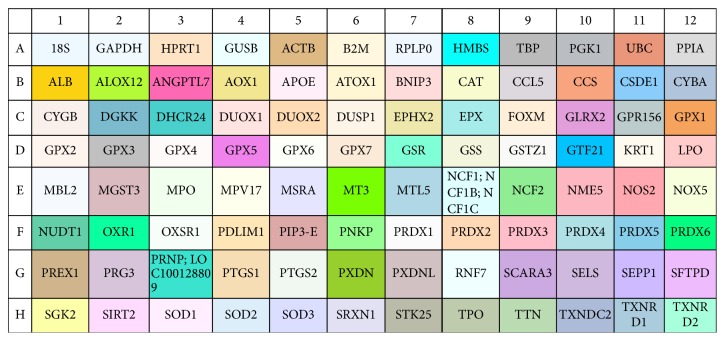
Gene symbols of 96-well plate. For real-time PCR genes are analyzed by TaqMan Array.

It has been reported that the expression of three important genes (TNF, NF*κ*B1, and Bcl-2), involved in cell survival, was modulated in mice fed with LBB suspension [42]. Based on these results, to investigate this aspect in a more exhaustive manner, quantitative real-time PCR-array analysis, with a panel of 96 genes involved in oxidative stress, proliferation, apoptosis, and cancer, was performed in HepG2 cells (Figure 5). We used 1800 *μ*g/mL concentration of LBB extract to test gene expression in HepG2 cell line because this concentration did not change cell viability and genotoxicity.

**Figure 6:**
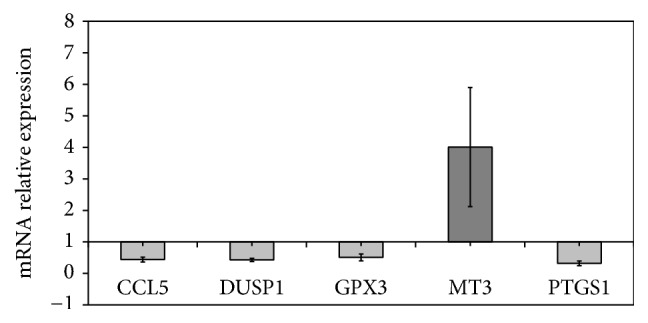
Relative expression of different genes in HepG2 cells after treatment with LBB extract (1800 *μ*g/mL) and normalized with GAPDH housekeeping gene. Results are expressed as the mean ± SD of three independent experiments.

For real-time PCR, mRNA levels were normalized using GAPDH as internal control. The results show that few specific genes are modulated by LBBs. As shown in Figure 6, where the gene expression is referred to that of untreated cells, CCL5, DUSP1, GPX3, and PTGS1 genes are downexpressed by 0.44 ± 0.08%-, 0.43 ± 0.05%-, 0.52 ± 0.12%-, 0.33 ± 0.08%-fold, respectively, and MT3 gene is overexpressed by about 4.0 ± 1.89%-fold. CCL5 is an 8 kDa protein classified as a chemotactic cytokine or chemokine that exerted protumoral effects on human hepatoma cells through its G protein-coupled receptor, CCR1, and is involved in HepG2, Hep3B, and Huh7 human hepatoma cell migration, invasion, or spreading induced by the chemokine [43]. Dual-specificity phosphatases 1 (DUSP1) belong to a protein family responsible for dephosphorylating threonine/serine and tyrosine residues on their substrates; it is associated with different kinds of cancers and with an increased risk of metastasis and shorter overall survival [44]. In HepG2, DUSP1 prevents the apoptotic effect which is mycotoxin-induced [45]. GPx-3 is a selenoprotein belonging to the glutathione peroxidase family upregulated in HepG2 cells, indicating its role in the development of liver carcinogenesis [46]. Prostaglandin H synthase 1 (PTGS1) is implicated in colorectal carcinogenesis [47] and it is considered a good target for cancer therapy [48]. Metallothionein 3 (MT3) is considered a putative tumor suppressor gene [49]. Decreased expression of MT3 has been found in gastric cancer, esophageal adenocarcinoma, and squamous cell cancer [50, 51].

Therefore, downexpression of genes involved in tumor migration and invasion (CCL5), in increased risk of metastasis and antiapoptotic signal (DUSP1), and in carcinogenesis (GPx-3 and PTGS1) together with overexpression of tumor suppressor gene (MT3) suggests that Umbrian LBBs play an anticancer role. However, potential cancer-suppressive effects of LBBs should be further evaluated in* in vivo* and* in vitro* experiments. If you consider the high antioxidant activity of LBBs, it is possible to suppose that their potential anticancer role* in vitro* could be due to the high content of polyphenols. In fact, Chen et al. described a correlation between flavonoids and antiproliferative activities of* Rhamnus davurica* [52] and Xia et al. demonstrated a potential antihepatocellular carcinoma agent of flavonoids, using HepG2 cell line [38]. Among flavonoids, gallic acid is known to be an anticancer agent since it reduces cell survival, proliferation, and invasion in PC3 cells by downregulating IL-6 with consequent reduction of pSTAT3, pERK1/2, and pAKT signaling proteins [53]. At the moment the phytochemical composition of LBBs from Umbria is unknown. For further experiments it will be useful to clarify this point.

## 4. Conclusion

In conclusion the* Lycium barbarum* plant, originally cultivated in East Asia, has Umbrian environment adaptability. This could be due to the ability of the plants to learn from experience and to memorize previous experiences in order to optimize the acclimation to environmental stresses. This behavior is considered a form of intelligence of the plants [41]. The overall results show, for the first time, that the LBBs cultivated in Umbria have not only high antioxidant properties, but also a significant antigenotoxic effect. Finally LBBs appear to regulate the expression of genes involved in tumor progression and metastasis. However, prospective cancer-suppressive effects of LBBs should be further evaluated in* in vivo* and* in vitro* experiments.

## Abbreviations

AAPH: 2,20-Azobis(2-methylpropionamide) dihydrochloride
BT: 1,2,4-Benzenetriol
cDNA: Complementary DNA
DAPI: 4′,6-Diamidine-2′-phenylindole dihydrochloride
DW: Dry weight
FBS: Fetal Bovine Serum
HepG2: Human Caucasian hepatocyte carcinoma
LMPA: Low-melting-point agarose
LBBs: * Lycium barbarum* berries
MEM: Eagle's Minimum Essential Medium
MTT: 3-[4,5-Dimethyl-2-thiazolyl]-2,5-diphenyl-2-tetrazolium bromide
NMPA: Normal-melting-point agarose
ORAC: Antioxidant assay by oxygen radical absorbance capacity
PBS: Phosphate-buffered saline
RTqPCR: Real-time polymerase chain reaction
TE: Trolox equivalents
TPC: Total phenolic content
Trolox: 6-Hydroxy-2,5,7,8-tetramethylchroman-2-carboxylic acid
Tris: Tris(hydroxymethyl)-aminomethane.
